# A Prevention-Focused Geospatial Epidemiology Framework for Identifying Multilevel Vulnerability Across Diverse Settings

**DOI:** 10.3390/healthcare14020261

**Published:** 2026-01-21

**Authors:** Cindy Ogolla Jean-Baptiste

**Affiliations:** Descendants of Africa Pioneering Innovations (DAPI), Belton, TX 76513, USA; caogolla@gmail.com; Tel.: +1-6096659117

**Keywords:** geospatial epidemiology, interpersonal violence, environmental risk, spatial analytics, prevention informatics, health equity

## Abstract

**Highlights:**

**What are the main findings?**

- Geospatial epidemiology uncovers spatially patterned vulnerabilities driven by ecological, structural, and built-environment determinants.
- The proposed architecture translates multilevel data into localized risk signatures for precision prevention strategies.

**What are the implications of the main findings?**

- Spatial intelligence enables earlier identification of risk, context-aware clinical screening, and targeted community-level interventions.
- Integrating geospatial analytics across systems helps overcome systemic data fragmentation, advancing equitable, context-responsive public health action.

**Abstract:**

**Background/Objectives**: Geographic Information Systems (GIS) offer essential capabilities for identifying spatial concentrations of vulnerability and strengthening context-aware prevention strategies. This manuscript describes a geospatial architecture designed to generate anticipatory, place-based risk identification applicable across diverse community and institutional environments. Interpersonal Violence (IPV), one of several preventable harms that benefit from this spatially informed analysis, remains a critical public health challenge shaped by structural, ecological, and situational factors. **Methods**: The conceptual framework presented integrates de-identified surveillance data, ecological indicators, environmental and temporal dynamics into a unified spatial epidemiological model. Multilevel data layers are geocoded, spatially matched, and analyzed using clustering (e.g., Getis-Ord Gi*), spatial dependence metrics (e.g., Moran’s I), and contextual modeling to support anticipatory identification of elevated vulnerability. **Framework Outputs**: The model is designed to identify spatial clustering, mobility-linked risk patterns, and emerging escalation zones using neighborhood disadvantage, built-environment factors, and situational markers. Outputs are intended to support both clinical decision-making (e.g., geocoded trauma screening, and context-aware discharge planning), and community-level prevention (e.g., targeted environmental interventions and cross-sector resource coordination). **Conclusions**: This framework synthesizes behavioral theory, spatial epidemiology, and prevention science into an integrative architecture for coordinated public health response. As a conceptual foundation for future empirical research, it advances the development of more dynamic, spatially informed, and equity-focused prevention systems.

## 1. Introduction

Many forms of preventable risk, including hazardous environmental exposures, IPV, crime transportation-related vulnerabilities, and protective social resources, follow spatial patterns that are not readily observable in traditional surveillance systems. Public health responses have historically relied on retrospective reporting and non-geographic datasets constraining health systems and community partners to reactive approaches. Consequently, systems miss critical opportunities for anticipatory prevention in high-risk environments, allowing harm to escalate.

Interpersonal violence (IPV) imposes significant physical, psychological, and economic consequences on survivors, families, and communities, remaining a central concern in public health research and practice [1]. More importantly, IPV, particularly sexual violence, is unequivocally a healthcare issue with well-documented and substantial downstream clinical consequences, including chronic pain, traumatic stress, reproductive and obstetric complications, substance use escalation, and elevated long-term healthcare utilization [2]. These sequelae constitute a significant portion of the preventable clinical burden necessitating effective, coordinated health-promotion models. Unfortunately, a persistent divide between clinical practice and population-health systems has led to fragmented data streams and missed opportunities for coordinated care [3]. These organizational silos, often counterintuitive to the goal of health equity, create a form of “structural blindness” that prevents health systems from seeing the very patterns of risk they are tasked with preventing.

Geographic Information Systems (GIS) have transformed disease surveillance, environmental monitoring, and chronic condition management by enabling precise identification of spatial clustering, ecological disparities, and opportunity structures that contribute to population health outcomes [4,5]. Despite this demonstrated utility, GIS remains underutilized in prevention science. The novelty of this architecture lies not in the invention of new statistical techniques, but in the integrative synthesis and applied translation of existing methods for prevention-oriented use. The framework advances the field in three ways:

- First, it operationalizes established spatial epidemiology methods within an anticipatory prevention workflow designed to identify risk before harm escalates;
- Second, it provides a concrete blueprint for clinical and health system integration, demonstrating how spatial intelligence can be embedded into routine decision-making processes such as trauma screening, discharge planning, and service coordination; and
- Third, it advances an ethical reframing of data fragmentation, positioning siloed data systems not merely as technical limitations, but as a form of structural blindness that public health and healthcare systems have an obligation to address.

While illustrated with IPV, the framework is intentionally broad, integrating surveillance indicators with multilevel socio-ecological and environmental determinants into a unified spatial epidemiology model [6].

## 2. Materials and Methods

This section details the grounded process used to construct the multilevel geospatial epidemiology framework. The method follows a multi-stage analytic pipeline designed to translate raw data into actionable intelligence, moving from cluster detection to contextual analysis and finally to operational applications.

### 2.1. The Geospatial Imperative in Prevention

Most publicly available datasets relevant to preventable harms lack the spatial identifiers necessary for precision prevention. For example, most national public-use files omit neighborhood, tract, and block-group identifiers, as well as environmental exposures and mobility contexts, all of which are essential for contextual risk assessment [7]. Secure-access administrative and institutional datasets contain these geographic markers but require secure access protocols and institutional review board approval. Spatial identifiers are essential for linking preventable harms to social determinants of health (SDOH), environmental exposures, and neighborhood dynamics [8]. Without geographically explicit data, analysts cannot detect micro-clusters, identify hot spots, evaluate ecological predictors, or integrate spatial risk information into precision prevention workflows across clinical and community systems.

#### 2.1.1. Theoretical Foundations

We acknowledge the wider spectrum of safety-related outcomes beyond IPV that are relevant to precision prevention, including exposure to hazardous environments, opportunity-driven harms, and inequities in access to protective social conditions. Three behavioral theories are foundational to this framework:

- Routine Activities Theory (RAT) posits that preventable harms from violence to accidental injury occur when opportunity structures align with inadequate guardianship and environmental vulnerabilities; when a motivated individual, a suitable target, and the absence of a capable guardian converge in space and time [9]. Although traditionally applied to crime, the core framework applies broadly to public health contexts involving environmental exposure, transportation risk, substance-related harm, and unsafe social or physical environments. Our framework operationalizes these constructs using contextual data such as mobility corridors, nighttime activity zones, guardianship indicators like lighting, and location-specific environmental hazards to proxy these situational markers. However, when fragmented systems prevent integration of this data with health and justice records, the underlying “opportunity structure” for harm remains invisible, leaving public health responses perpetually reactive rather than anticipatory.
- Social Disorganization Theory (SDT) suggests that community-level structural factors such as residential instability, economic deprivation, and weakened institutional capacity diminish collective efficacy and heightened vulnerability [10]. When translated into spatial indicators, these community stressors help predict risk clustering by identifying neighborhoods that may require enhanced clinical screening, community engagement, and targeted prevention strategies. Data fragmentation, however, prevents analysts from distinguishing between a series of isolated incidents and the true structural hotspots predicted by SDT, fundamentally obscuring where neighborhood-level interventions are most needed.
- Lifestyle Exposure Theory (LET) explains how social roles and institutional affiliations, such as being a college student or a service member, lead to specific lifestyles that can increase exposure to high-risk environments [11]. This theory is particularly salient for institutional safety analysis. When identifiers for military or university affiliation are suppressed or unlinked from health and justice data, the specific risk signatures associated with these institutional lifestyles are erased. This creates an “institutional blind spot” that prevents the development of tailored, context-aware prevention strategies within these defined populations.

Additional constructs can be contextually incorporated, for example, Environmental Criminology extends beyond violence to emphasize how built-environment features and routine spatial behaviors influence the geographical distribution of preventable harms across communities [12]. Factors such as street connectivity, land-use patterns, visibility, and service accessibility shape how individuals encounter risk or protection within their everyday environments.

#### 2.1.2. Value of GIS for Anticipatory Prevention and Health Equity

GIS is essential for translating the theoretical need for data integration into a practical strategy for prevention monitoring. By geocoding and layering indicators of neighborhood disadvantage, environmental exposures, and population mobility, GIS allows practitioners to visualize how structural inequities discussed in theory manifest geographically and to detect emerging clusters of risk and address spatially patterned vulnerabilities that contribute to multiple forms of preventable harm before they evolve into acute events. These spatial insights reveal risk patterns that are often invisible in conventional datasets, enabling more precise allocation of prevention resources and more equitable deployment of interventions across communities [13,14].

In practice, GIS enhances anticipatory prevention by illuminating the environmental, temporal, and social conditions that elevate the likelihood of harm. For example, spatial analyses can identify high-risk microenvironments such as areas with poor lighting, limited surveillance, high transit flow, or concentrated commercial activity, which may influence both the opportunity for harm and the capacity for protective guardianship. These geospatial patterns provide the empirical grounding needed to design targeted environmental modifications, cross-sector outreach, and clinical screening protocols that account for contextual risk rather than relying solely on individual-level characteristics [12].

Importantly, integrating spatial epidemiology into prevention serves the goal of health equity. Rather than simply identifying structurally marginalized neighborhoods where cumulative disadvantage increases vulnerability to preventable harm. Spatial epidemiology reveals where service gaps, environmental stressors, and systemic disinvestment intersect, guiding the distribution of prevention resources in ways that counterbalance longstanding inequities [14]. When integrated with behavioral theory and the SEM, geospatial analysis helps clarify how structural and situational determinants converge to produce differential risk across communities, reinforcing the need for evidence-driven, place-based public health action.

### 2.2. Prevention-Focused Spatial Epidemiology Framework

**Figure 1:**
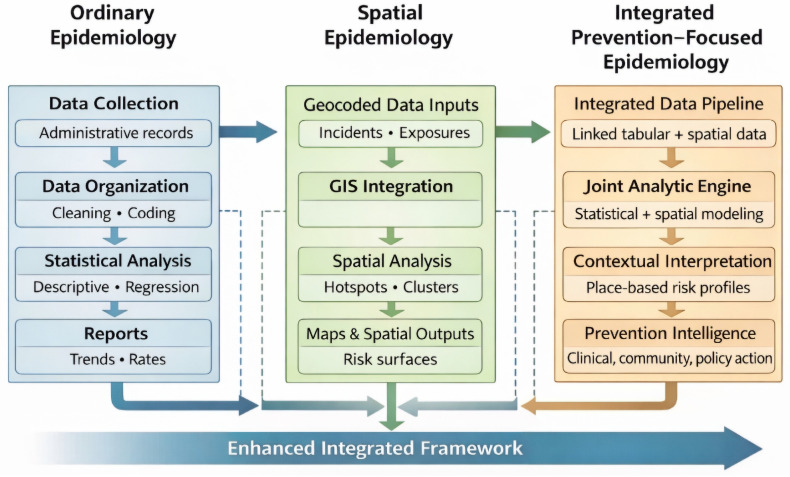
This figure illustrates the five-stage workflow of the architecture, moving from the integration of multisector data and its spatial alignment to rigorous spatial pattern analysis. The outputs from this process generate dynamic risk and vulnerability surfaces that are then used to guide the deployment of targeted clinical, community, and environmental prevention strategies. (*A conceptual, non-operational illustration*).

**Figure 2:**
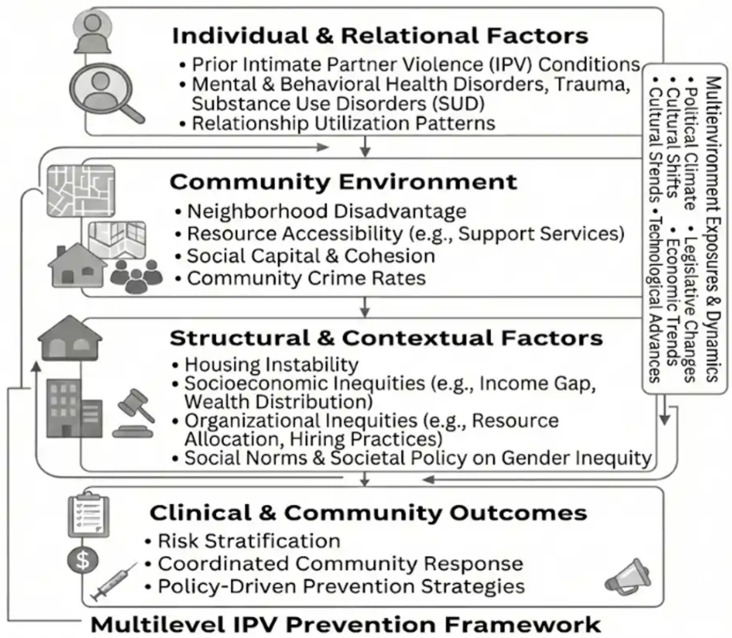
A multilevel conceptual model illustrating how determinants across individual, relational, community, and structural levels are synthesized to explain the emergence of spatially patterned vulnerability and inform precision prevention strategies. (*A conceptual, non-operational illustration*).

**Figure 3:**
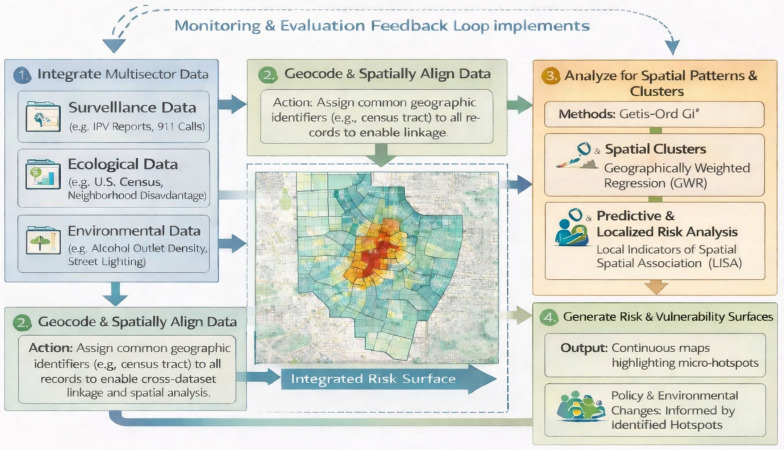
The figure depicts a five-stage workflow, starting with (1) integration of multisector data and (2) geocoding to (3) rigorous spatial pattern analysis, to generate (4) a final, integrated risk and vulnerability surface, which can then be used to guide targeted prevention strategies. Solid arrows represent the primary sequential workflow. Dashed arrows indicate iterative feedback loops. (*A conceptual, non-operational illustration*).

A comprehensive spatial epidemiology framework for prevention requires the integration of geographic, ecological, environmental, and temporal indicators across multiple levels of influence. The conceptual diagrams (Figure 1, Figure 2 and Figure 3) presented in this section illustrate the multilevel spatial integration process, hotspot modeling workflow, and neighborhood-level risk surface generation that underpin the analytic approach described herein. Figures were conceptually developed by the author and refined using generative artificial intelligence tools. These figures should be interpreted as conceptual, non-operational illustrations of the proposed framework.

#### 2.2.1. Multilevel Data Architecture for Spatial Prevention Modeling

The framework’s architecture is built on three primary categories of data, which are geocoded to geographically meaningful units (e.g., census tracts) to detect neighborhood-scale variation:

1. Incident and Surveillance Data: This layer includes de-identified reports of harm, such as IPV incidents, substance-related emergency calls, or other safety records. When available, secure-access administrative datasets can provide additional temporal and situational markers, including the time of day and contextual circumstances of an event, which are critical for detecting fine-grained risk dynamics [7,15].
2. Ecological Indicators: This layer quantifies underlying structural risk by incorporating community-level structural factors from sources like the U.S. Census. Applying key indicators reflecting economic disadvantage, residential mobility, and weakened collective efficacy illuminates underlying structural risk [9].
3. Environmental Exposures: This layer provides situational context by mapping features of the built environment that influence vulnerability such as zoning regulations, commercial density, transportation networks, and guardianship indicators such as street lighting, the proximity to alcohol outlets and vacant lots [12].

#### 2.2.2. The Barrier: A Fragmented Data Landscape

Despite the utility of spatial epidemiology, prevention-relevant data systems remain highly fragmented, impeding precision risk modeling and weakening integrated prevention efforts. This problem manifests in two distinct ways:

- Geographic Obscurity. This problem stems from a lack of standardized geographic identifiers across datasets. To preserve confidentiality, data are often intentionally truncated to a zip code or county level, a practice that severely limits their utility for the neighborhood-level analysis required for cluster detection. This issue is particularly acute in many public-use datasets, which frequently omit block group or tract identifiers altogether, thereby preventing the crucial linkage of incidents to environmental exposures or SDOH [7].
- Systemic Silos. Health, justice, and contextual, for instance behavioral and social service records are typically maintained in separate systems that seldom interoperate, a problem compounded by inconsistent variable definitions and variations in data quality. This fragmentation is especially pronounced within large organizations, such as academic institutions and military installations, where incident reporting, behavioral health encounters, and environmental monitoring records exist in disconnected databases. This makes it nearly impossible to connect structural or situational context to prevention and safety outcomes [16].

#### 2.2.3. The Solution: Pathways to Data Integration and System Modernization

Addressing geospatial surveillance limitations requires deliberate investments in data interoperability, standardized geographic identifiers, and coordinated administrative governance. This framework addresses the barrier of fragmentation by proposing a deliberate investment in an integrated data ecosystem achieved through two complementary pathways:

- Governance and Standardization. The first pathway is establishing a common operational foundation. This involves creating formal data-sharing agreements and most critically mandating the routine incorporation of standardized geographic identifiers across all relevant datasets. This enables data to be reliably aligned and compared, forming the bedrock of any multi-sector analysis [3,17].
- Technology and Infrastructure. The second pathway is leveraging modern technology. Secure-access research infrastructures, often called “data enclaves,” allow analysts to work with restricted microdata containing detailed geographic identifiers while maintaining strict confidentiality [18]. This can be paired with advances in administrative informatics that support the near real-time integration of data, enabling dynamic, space-time analyses that dramatically enhance situational awareness [19,20].

The pathways described above enable the comprehensive, prevention-focused workflow illustrated in Figure 1.

Figure 1 Conceptual Workflow for the Prevention-Focused Spatial Epidemiology Framework (*A conceptual, non-operational illustration*).

### 2.3. Analytic Framework

This framework incorporates spatial analytic tools to translate multilevel data into interpretable spatial patterns, highlighting inequities, and illustrating how contextual forces shape the geographic expression of risk.

#### 2.3.1. Analytic Stage 1: Detection of Spatial Clustering and Spatial Dependence

The first analytic stage is to move beyond simple case counts and provide rigorous evidence of non-random geographic distribution. Preventable harms, including IPV, often concentrate in identifiable microenvironments such as nightlife districts, university border areas, transitional housing corridors, and communities near military installations. These locations are shaped by mobility flows, limited guardianship, commercial density, and environmental features that influence risk. To identify these concentrations with statistical confidence, spatial analytic tools should encompass both visualization and validation. Visualization employs tools such as kernel density estimation, while validation is achieved through Global Moran’s I, Local Indicators of Spatial Autocorrelation (LISA), and Getis-Ord Gi* statistics to formally detect clustering and assess spatial dependence to provide rigorous evidence of non-random geographic distribution [20,21]. These analyses reveal micro-hotspots where prevention resources may have maximal impact. The use of Geographically Weighted Regression (GWR) then allows for an assessment of how predictor relationships, (e.g., the influence of alcohol outlet density or inadequate lighting), vary across communities, strengthening the evidentiary basis for targeted, place-specific prevention strategies [22]. It is critical, however, to account for potential biases such as the Modifiable Areal Unit Problem (MAUP) and the effects of spatial autocorrelation during this stage [15] because these biases can artificially create or hide risk clusters depending on the geo-graphic boundaries (zip codes, police precincts, or census tracts) chosen for the analysis.

#### 2.3.2. Analytic Stage 2: Integration of the SEM

Moving from “where” to “why,” this second analytic stage extends far beyond individual characteristics integrating socioecological conditions. The workflow first incorporates community and structural factors that profoundly shape patterns of vulnerability across communities such as neighborhood disadvantage, housing instability, inequitable access to protective resources, and weakened social capital resources. It then layers on more proximal situational determinants by modulating these baseline risks with temporal dynamics, including nighttime activity, event-driven mobility to track seasonal cycles, and situational determinants of harm. Integrating these multilevel determinants produces multidimensional vulnerability surfaces that can identify both where and when risk intensifies. As conceptually illustrated in Figure 2, resulting surfaces reveal micro-zones where ecological stressors and environmental exposures converge, providing an evidence base for precision prevention rooted in multisector coordination that targets hotspots identified in the previous stage.

Figure 2 Multilevel Conceptual Model Linking Socio-Ecological Determinants to Spatially Patterned Risk. (*A conceptual, non-operational illustration*.)

### 2.4. Spatial IPV Framework and Conceptual GIS Workflow

The conceptual GIS workflow (Figure 3) demonstrates how multilevel contextual information can be integrated to identify micro-hotspots and generate neighborhood-level risk surfaces for predictive analytics. The process begins with foundational geographic units which may include census boundaries, administrative zones, institutional districts, or other locally relevant polygons that anchor the analysis. The second step overlays incident locations or density-adjusted counts that reveal clustered patterns of harm. The third panel incorporates structural and environmental predictors drawn from community indicators, organizational administrative records, or population-level surveys. The final step incorporates structural and environmental predictors drawn from community indicators or administrative records. This process converts multiple data layers into a continuous risk surface that reflects the complex interplay between events and the contextual conditions that influence vulnerability.

Figure 3 Multilevel Conceptual GIS Workflow for Integrated Risk Surface Generation. *(A conceptual, non-operational illustration).*

### 2.5. Applications of the Spatial Workflow Across Settings

This spatial workflow demonstrates its adaptability by applying its core principles across diverse environments. In each setting, the framework is designed to overcome common data fragmentation challenges and produce actionable intelligence, supporting the identification of micro-hotspots, structural vulnerability, and environmental conditions that elevate risk or escalation of harm.

#### 2.5.1. National and Population Level Analysis

National or Population-Level Datasets: At large geographic scales, the framework can be used with national datasets like the National Crime Victimization Survey (NCVS) Census tracts or block groups map variability in IPV indicators (e.g., victimization survey responses or reporting patterns). When integrated with socioeconomic vulnerability, residential mobility, and resource access data, they provide insight into regional patterns of risk intensity and chronicity, highlighting communities facing disproportionate burdens [7].

#### 2.5.2. Clinical and Healthcare Settings

The framework most directly strengthens trauma-informed clinical decision-making by embedding spatial context into routine screening workflows. This is critical because exposure to violence is a known driver of long-term sequelae, from chronic injury and pain to lasting mental health effects such as post-traumatic stress [2]. Geocoded residential information can signal environmental risks not captured in a patient’s self-report, particularly for patients living in structurally marginalized or environmentally hazardous areas. Integrating spatial risk indices into electronic health records (EHR) enables a shift from reactive care to proactive, spatially informed screening and decision-making [23]. This process improves the identification of individuals who may require additional assessment and safety planning. Consequently, it guides clinicians in safe discharge planning and linkage to trauma-informed community resources, ensuring prevention strategies align with the structural realities of the populations served [3,24].

#### 2.5.3. Municipal and Community Settings

At the local level, the framework can map IPV calls-for-service, emergency department encounters, or local agency data. Integrating socioeconomic stress, neighborhood disorder, housing instability, resource scarcity, and alcohol outlet density highlights micro-hotspots where contextual disadvantage magnifies risk and limits service access. However, these efforts are often hampered when health and justice data remain unlinked, a limitation that has led to active calls for greater data integration to achieve a true public health approach to violence prevention [25].

#### 2.5.4. Institutional Settings

Integrating indicators of institutional strain and access to services helps identify spatial concentrations of IPV near high-stress work areas or transitional housing spaces

- Military: The framework can examine how operational stressors, population turnover, isolation patterns, or duty-related schedules relate to IPV occurrence using installation districts, housing zones, or unit areas as the unit of analysis. Yet, analysts often face significant barriers in tracking personnel across different commands or linking on-base and off-base incidents, a data fragmentation issue noted in governmental audits that hinders effective prevention planning [26].
- College and Campus Settings: Similarly, on college campuses, the framework allow exploration of spatial clustering of dating violence, coercive control, and stalking using campus sectors, residence halls, or security patrol zones as the unit of analysis [15]. Combining campus climate indicators and environmental vulnerabilities can reveal concentrated risk near social hubs or low-visibility pathways where tailored prevention may be most impactful. However, such analyses are often limited by the same systemic silos seen in other large institutions, where law enforcement data is separated from student conduct and health records, masking the true spatial patterns of risk [16].

## 3. Results

Although interpersonal violence serves as an illustrative case, the framework applies broadly to other spatially patterned public health challenges, and community safety concerns. By combining spatial analytics with contextual indicators, organizations can adopt earlier, more targeted, and more equitable prevention strategies that reflect *lived* contextual realities [19,20]. The results of this framework are its specific applications across clinical, community, and institutional domains. The following sections detail three primary capabilities: (1) enhancing spatially informed clinical decision support, (2) fostering cross-sector coordination through shared intelligence, and (3) guiding environmental and situational prevention strategies.

### 3.1. Enhancing Spatially Informed Prevention and Clinical Decision Support

A primary application of this framework is its integration into clinical decision support processes. Geocoded residential information and neighborhood-level indicators reveal contextual risks not captured through individual self-report, especially for patients in structurally marginalized or environmentally hazardous areas. Integrating these spatial risk indices into existing screening or triage systems allows practitioners to recognize these contextual factors during safety assessments, and referral processes particularly for the known sequelae of violence like chronic injury or post-traumatic stress [24].

### 3.2. Fostering Cross-Sector Coordination and Shared Prevention Intelligence

The framework also provides a mechanism for strengthening collaboration across traditionally siloed sectors (e.g., healthcare systems, public health agencies, social services, municipal departments, universities, and military installations) by providing a shared view of geographically patterned vulnerability. Unified geospatial dashboards, and spatially linked datasets can reveal emerging clusters, service gaps, and resource disparities that might otherwise go unnoticed, supporting coordinated action among partners [3]. In institutional settings like military, these capabilities also reinforce readiness, environmental safety planning, and geographically targeted interventions [26].

### 3.3. Environmental and Situational Prevention Strategies

Finally, spatial analysis provides actionable guidance for modifying the physical and situational environments that influence exposure to preventable harm. Geographic patterns of risk can inform interventions such as improved lighting, enhanced guardianship, optimized transit routing, and adjustments to land use or alcohol outlet density. Evidence demonstrates that such targeted environmental modifications can meaningfully reduce public health harms by altering the opportunity structures that facilitate risk [9,12].

## 4. Discussion

The primary contribution of this conceptual framework is providing an integrative blueprint to bridge the longstanding divide between clinical practice and population health. It dictates why data integration is a structural imperative for effective prevention, revealing the specific mechanisms of risk that remain invisible when health and justice data are kept in silos. At its core, this proposed architecture shifts prevention from individual-only assessments toward a more comprehensive display of multilevel determinants of spatially patterned vulnerability.

### 4.1. Principal Contribution and Implications

The primary implication of this framework is its ability to create a multi-layered prevention system that addresses both individual and community conditions to reveal inequities not apparent in individual-level analyses alone. The framework’s value is realized by translating interpretable spatial intelligence into distinct forms of action: it aligns clinical outreach with the structural realities that shape patient harm, guides data-driven modifications to the physical environments that create risk and enables organizations to collectively address risk environments through shared spatial intelligence. When combined with upstream structural interventions, the strategies enabled by this framework create a multi-layered, anticipatory and context-aware prevention architecture [27].

### 4.2. Ethical Considerations and Data Governance

Geospatial analyses of IPV require robust ethical safeguards due to the sensitivity of spatially identifiable data. Responsible implementation demands a multi-pronged governance strategy:

1. Data Protection: Strict de-identification protocols and aggregation thresholds must be paired with tiered access controls within secure data enclaves accessible only to analysts with institutional review board approval [18]. Modern privacy-preserving machine learning techniques can provide further layers of protection [28].
2. Preventing Bias: To ensure spatial models do not stigmatize communities or reinforce historical inequities, implementation should include Participatory GIS (PGIS) methods where residents help contextualize data [3]. Furthermore, algorithms must be audited for fairness to evaluate whether they disproportionately assign risk to structurally marginalized populations.
3. The Ethical Imperative for Integration: An exclusive focus on the ethics of data use overlooks the profound ethical consequences of inaction. The systemic data fragmentation described throughout this paper creates a form of “structural blindness” that prevents public health systems from seeing and acting upon known risk mechanisms [16]. Therefore, there is an ethical imperative for integration, as failing to build the systems necessary to prevent harm perpetuates an inequitable status quo.

### 4.3. Limitations and Future Directions

This framework is presented as a direct response to longstanding calls for greater integration between public health, clinical care and institutions to address complex health challenges [3]. We acknowledge several limitations and future directions.

#### 4.3.1. Limitations

First, this manuscript presents a conceptual and methodological framework and does not include empirical validation. Secondly, its effectiveness depends on overcoming the pervasive structural barrier of data fragmentation that defines the current public health landscape. Third, it is also critical to reiterate that, like all spatial epidemiology, this approach identifies statistical associations and dependencies rather than definitive causal mechanisms [14]. Finally, we acknowledge that the practical implementation of this framework depends on significant institutional investments in data infrastructure and a skilled analytic workforce which may vary across different geographic and cultural contexts.

#### 4.3.2. Future Directions

This framework provides a foundation for a multi-pronged research and implementation agenda:

1. Empirical Validation: The immediate next step is its application to high-resolution, restricted datasets to assess its performance and validity.
2. Advocacy for Modernized Data Ecosystems: A critical long-term goal is to advocate for the development of integrated data ecosystems. This does not necessarily require centralizing all data; cutting-edge approaches like federated learning offer a powerful model for building collaborative analytics across siloed health systems while preserving patient privacy [29].
3. Integration of Explainable AI (XAI): Future iterations could benefit from integrating XAI tools to improve model transparency and help stakeholders understand the complex drivers of risk, thereby increasing trust and adoption [30].

## 5. Conclusions

The persistent fragmentation between clinical practice, public health surveillance and community systems has created a form of “analytic paralysis,” leaving prevention efforts fragmented and reactive. This manuscript presents an integrated geospatial framework as a direct response to this challenge. to strengthen prevention across clinical, public health, and community systems, aligned with contemporary precision public health priorities that emphasize actionable, high-resolution data to guide equitable and timely interventions [6]. By synthesizing behavioral theory with spatial epidemiology, the framework offers a practical blueprint for translating siloed data into actionable, place-based intelligence.

By embedding spatial insights across clinical and community domains, organizations can strengthen their capacity to anticipate risk and reduce preventable harm. The architecture established here provides a foundation for future empirical validation using high-resolution data and, more importantly, outlines a clear pathway toward coordinated, context-aware, and equity-oriented prevention systems.

## Data Availability

No new data were created or analyzed in this study.
